# Age and gender patterns in health service utilisation: Age-Period-Cohort modelling of linked health service usage records

**DOI:** 10.1186/s12913-023-09456-x

**Published:** 2023-05-12

**Authors:** Koen Simons, Owen Bradfield, Matthew J. Spittal, Tania King

**Affiliations:** 1 Centre for Epidemiology and Biostatistics, Melbourne School of Population and Global Health,University of Melbourne, 207 Bouverie Street, 3010 Melbourne, Australia; 2 Centre for Health Policy, Melbourne School of Population and Global Health,University of Melbourne, Melbourne, Australia; 3 Centre for Mental Health, Melbourne School of Population and Global Health,University of Melbourne, Melbourne, Australia; 4 Centre for Health Equity, Melbourne School of Population and Global Health,University of Melbourne, Melbourne, Australia

**Keywords:** Age-period-cohort, Health service utilisation, Linked data

## Abstract

**Background:**

Health service utilisation changes across the life-course and may be influenced by contextual factors at different times. There is some evidence that men engage less with preventive health services, including attending doctors’ clinics, however the extent to which this varies temporally and across different age groups is unclear. This study aimed to describe age or cohort effects on engagement with GPs among employed mothers and fathers in Australia, and differences in these trends between men and women.

**Methods:**

We linked data from the ‘Growing up in Australia: The Longitudinal Study of Australian Children’ with administrative health service records from Medicare. We used a small-domain estimation Age-Period-Cohort method to describe patterns in health service use among working-age male and female parents in Australia while adjusting for employment status and controlling for time-invariant factors. Our small-domain method assumes a smooth response surface of Age, Period and Cohort.

**Results:**

Male parents have lower health service engagement than women of the same age at the same time period. Men’s pattern of health service use across time is likely explained entirely by ageing. That is, we find that patterns in health service utilisation among men are largely driven by age effects, with no evidence of periods or cohort effects in health service engagement for men between 2002 and 2016.

**Conclusions:**

Differences in health service utilisation between male and female parents at all age-period-cohort combinations highlight a need for more research to examine the extent to which this level of health service use among Australian men meets men’s health needs, as well as barriers and enablers of health service engagement for men. Absence of evidence for period effects suggests that there is little shift in gendered patterns of health service utilisation during the observed period.

**Supplementary Information:**

The online version contains supplementary material available at 10.1186/s12913-023-09456-x.

## Background

Gendered patterns in health are well-established. Women are known to have higher rates of illness than men, they have higher rates of disability and their self-rated health is typically lower than that of men [1]. Despite this, women of all ages have lower death rates than men, and life expectancy for women is higher than that of men in most developed countries [2, 3]. A similar paradox is also observed in relation to mental health outcomes: while women typically have higher rates of mental illness than men, men are more likely to die by suicide [4].

A range of factors has been proposed to explain men’s higher mortality. Biological factors include telomere length, immune functioning, protective effects of oestrogen, and protection from oxidative stress [5]. Given that these factors are biological sex, they are not modifiable. Behavioural factors are also implicated in men’s higher mortality. These factors are influenced by societal norms and expectations, and are therefore potentially modifiable. Men engage in more reckless driving and are more prone to excessive alcohol consumption and cigarette smoking [6, 7]. Such factors may partially explain the male preponderance for coronary heart disease, hypertension, metabolic disease, cancer, overweight and obesity [5].

Another societal factor that may contribute to this health paradox is health service use: it is theorised that men engage less with health services and seek help later than women, when treatment options are fewer, and prognoses are poor. In the US, men are less likely than women to attend doctors’ clinics or present to emergency departments [7]. Also in the US, women are more likely than men to engage with preventative healthcare services, even when accounting for antenatal and reproductive health services [8]. A Danish study using national registry data revealed an overall pattern of lower contact with medical services, but higher rates of hospitalisation and mortality for men [9]. This is suggestive of an overall pattern of behaviour in which men do not sufficiently engage in routine preventive health services, and seek help too late, when treatment options are limited.

Statistics in Australia mirror these global patterns: men’s engagement with mental health services is typically lower than that of women [10]. However, there is emerging evidence of a narrowing gap between women and men in mental health service use for mental and substance use disorders [11]. However, little is known of gender differentials in relation to broader non-mental health service utilisation in Australia. In particular, little is known of differences of patterns and differences in the extent to which men and women avail themselves of General Practitioner (GP) services in Australia. This represents a key gap knowledge, as understanding patterns in health service utilisation is vital for the continued delivery of health services that meet the needs of the population.

GPs are central to the delivery of health services in Australia. They are typically an individual’s first point of contact with health services and GPs are frequently responsible for the coordination or management of an individual’s treatment - including referrals to other specialist providers - and the provision of health advice and education. Importantly too, GPs see the majority of injured workers and are key gatekeepers to workers’ compensation and disability benefits [12]. In Australia, all non-hospital health care services listed on the Medicare Benefit Schedule (MBS), such as those provided by GPs, are subsidised by the Federal Government such that individuals only pay a proportion of the service cost [13]. Providers almost always bill Medicare directly for services, and many GPs claim directly (“bulk billing”) meaning that there is no need to advance the bill and reclaim later when out-of-pocket costs are zero [13]. As such, the direct costs of GP visits are negligible and should not be a barrier that complicates patterns in health service usage.

Health service utilisation changes across the life-course [14]. However, it is also likely that patterns of health service utilisation will change depending on broader contextual factors at different times (e.g. the availability and cost of particular services, or whether and when items are added to or removed from the MBS), as well as different birth-cohort effects, for instance through changes in attitude or differences in history of environmental exposures.

### The current study

Beginning with a cohort study of children and their families, we obtained additional administrative data at the individual level to clarify the relationship between gender and health service utilisation. The onset of parenthood is an important life-stage and is often associated with a divergence in roles, particularly in non-same-sex couples. The roles and behaviours adopted by heterosexual couples in early parenthood often remain entrenched along gendered lines. Examining patterns of health service utilisation among individuals in early stages of parenthood may identify age, period or cohort effects that drive health service utilisation trends, and thereby help to direct interventions. In this paper, we sought to examine patterns in health service utilisation across different ages and time from a longitudinal dataset of Australian parents using descriptive Age-Period-Cohort (APC) methods. We focussed on employed individuals for three reasons. First, the relationship between gender, unemployment and health is complex, and failing to adequately control for this in models will lead to bias. Second, given that in Australia, the majority of men and women with dependents work, employed parents represent a key study group. Third, workplaces may be important settings in which to intervene to encourage and support help seeking and health service engagement. The specific aims of this study were to:

1. Describe current trends in engagement with GPs among employed mothers and fathers in Australia.
2. Describe difference in these trends between men and women.
3. Examine any age or cohort effects on health service utilisation among these groups.

## Methods

### Datasource

Data was drawn from the ‘Growing up in Australia: The Longitudinal Study of Australian Children’ (LSAC). LSAC commenced in 2004, with 10,000 families enrolled in two groups: group B (‘birth’, aged 0/1 years at baseline) and group K (‘kindergarten’, aged 4/5 years at baseline) [15]. The LSAC study focusses on children and their families, capturing relevant data on a range of outcomes from their parents, carers and teachers, as well as the study children. Data is collected every two years, with the majority of survey responses collected in even years. In the seventh wave of data collection, 2016-2017, parents were asked to consent to having the historical medical data pertaining themselves linked to the LSAC (consent for linkage of their childrens’ data was sought earlier). This includes the Medicare Australia database [13], an administrative database collecting information on health service usage when the usage is wholly or partially paid by the public health system that is available for all Australian citizens.

### Medicare data

This paper focussed on the health service usage records of parents. As parents were asked to consent to linkage to their own Medicare data in wave seven, available data on parents’ health service usage is a subset of an otherwise prospective observational design. Some of the adults included in this study were not originally the parents of a child in LSAC, owing to changes in relationships and custody. To ensure consistency of parents across waves of data collection, we matched dates of birth, gender and study child id. This procedure also prevented confusion caused by relationship dissolution and formation, with low probability of errors. While we used the child identifier, this was solely as a means to link data about the parents, the focus of our research.

To determine the number of times a participant consulted with a GP in a calendar-year, we identified all relevant Medicare item-numbers from the data. We chose codes that the GPs use for billing Medicare, thereby excluding hospital procedures and services carried out by medical specialists other than GPs. The full list of MBS item numbers and their incidence in the data is listed in Supplementary Material Table 1. Records occurring on the same day or on consecutive days were considered as part of a single episode such that a person with recorded items on 01 January, 02 January and 03 January was counted as one episode, while a person with items delivered on 01 January, 02 January and 04 January was counted as two episodes. To control for health services associated with pregnancy and childbirth, we created a binary indicator for women, with 1 denoting the occurrence of any item relating to child-birth (ante- or peri-natal care including items 16400 to 16573, see Supplementary Material Table 1) that was recorded in the previous year or the year of the interview.

Health service utilisation data was restricted to time periods that parents reported complete information, e.g. a parent who reported employment in wave one and three but did not answer the employment question in wave two, contributed to the analysis of his or her GP visits in 2004-2005 and 2008-2009, but not the service usage of 2006-2007. Service usage data for 2017 was incomplete and therefore discarded for the entire sample.

### Sample and missing data

At baseline, 5107 and 4983 households were recruited for the B group (‘birth’, parents with a child aged 0/1 years at baseline), respectively K group (‘kindergarten’, aged 4/5 years at baseline). In group B and in group K, fewer male parents responded at baseline, resulting in 10% and 14% more female parents compared to male parents in group B, respectively K. All groups suffered a cumulative drop-out ranging 35% to 45% by wave 7. Relatively few new parents were introduced between waves 1 and 7: of parents responding in wave 7 approximately 10% of male parents and approximately 2% of female parents joined the study after baseline.

At wave 7, consent for record linkage was sought from participating parents. More than 80% of female parents consented in both B and K groups, however, only 56% to 58% of male parents consented to linkage. As such, the final sample size of female parents was larger than that of male parents, with similar sample sizes for both group B and group K: the final sample size is 2661 and 2590 female parents for the B group, respectively K group, and the corresponding sample sizes for men are 1570, respectively 1436. Supplementary Material Table 2 presents the flow chart for selection into the sample.

### Statistical analysis

We carried out descriptive APC analysis to examine patterns in health service utilisation among women and men. A priori, age (A), period (P) and cohort (C) effects are plausible in our data.

Age effects are those associated with the biological process of development and ageing: an infant is associated with different health and disease outcomes compared to an elderly person. The age of participants in our study ranges 20 to 66 years, as we are interested in the general working population, and it is universally acknowledged that there are noticeable differences across this range. In addition, such expectations may themselves affect the health-seeking behaviour of individuals as they age.

Period effects are what most would understand as historical events or influences (e.g. war, diagnostic standards, funding of health care, etc.) - events and circumstances that affect the population at a particular time. Our study has a relatively short span of time, 2002 to 2016, however this includes the 2008 Global Financial Crisis. In addition, the severity and impact of influenza-like illnesses is known to vary from year to year.

Cohort effects are those that influence a particular birth cohort, for example experiencing a famine during childhood will exert a particular effect on that cohort across the life-course. Events may have both immediate consequences and long-lasting consequences, for instance famine is a period effect at the time of the famine and a cohort effect as those who survived the famine may have different characteristics from those who were born after the famine. Although the study period itself is limited, the range of age at baseline is 18 to 50 years old and these are parents with a newborn or four year old child. The difference in birth-cohort is likely associated with differences in socio-economic position and that is likely to affect health and health-seeking behaviour.

The potential presence of all three effects poses a challenge: A = P - C and therefore the effect size estimates are completely confounded. All Age-Period-Cohort (APC) analyses require constraints - strong assumptions - for attribution albeit these are not always properly understood or stated explicitly [16]. Comparisons of estimates obtained from multiple studies are similarly difficult [17]. However, no strong assumptions are required to use the APC framework as a descriptive analytical tool for the questions ‘is there substantial variation in the population?’ and ‘which groups are most affected?’ While there is no unique attribution provided by the data itself, potential explanations for observed patterns can be judged by expert opinion.

With sufficiently large datasets, observed patterns require only to visualise the data. In smaller samples, noise is an additional nuisance that can be addressed by smoothing. Such statistical techniques do not assume that age, period and cohort are all additive and linear effects. We assume that these APC effects vary smoothly such that a person aged 25 years in 2006 is more similar to a 26 year old person in 2006 than to a 45 year old person in 2006 or a 25 year old person in 2016 [18]. This assumption allows small domain estimation of expected numbers of GP visits for each individual age-period-cohort combination: borrowing information from adjacent ‘domains’ provides estimates with reasonable precision even when there are few (or none) observations in a domain. To enable this, we included a smooth function *s*(, ) of birth-year $C_{i}$ and calendar-year *t* in the model. Specifically, we used tensor product splines to model the surface $s(C_i,t)$. As age is a trivial linear combination of birth-year and calendar-year, this effectively adjusts for the Age, Period and Cohort effects.

Our analysis assumes that the number of GP visits $y_{it}$ is a count process that is influenced by multiple observed and unobserved factors. Observed factors are age, calendar-year, gender and labour force status. The decision for health service utilisation depends on the individual’s health status as well as their health-seeking behaviour and neither are observed directly. As a first approximation, we assume that health-seeking behaviour is a time-invariant individual-level latent variable $u_i$ and a mixed model can be accommodated for such factors. The individual’s health status can be seen as a combination of time-invariant and time-dependent factors. Time-invariant factors such as genotype and early childhood exposures, are absorbed into the latent variable, while time-dependent factors are absorbed into the global APC effect, essentially assuming there are no within-person APC effects.

We are interested in the employed population but participants employment status (employed, unemployed, not in labour force) varies. Therefore we adjusted for this observed variable using dummy indices. We modelled the number of GP visits as a count variable and used the log-link, which is the canonical link function for a Poisson model. The complete model is:

1 $$\begin{aligned} \left\{ \begin{array}{ll} y_{it} &{} \sim \textrm{Poisson} (\mu _{it}) \\ \textrm{log} (\mu _{it}) &{} = \alpha + u_i + s(C_{i},t) + \beta _1 \textrm{nilf}_{it} + \beta _2 \textrm{unemployed}_{it} \\ u_i &{} \sim N(0,\sigma ) \end{array} \right.&\end{aligned}$$

Herein $s(C_{i},t)$ is a smooth effect of Cohort and Period. Dummy indices are used for not in labour force ($\textrm{nilf}_{it}$) and unemployed ($\textrm{unemployed}_{it}$), with the reference category employed parents. $u_i$ captures person-specific time-invariant, latent factors that influence health and health-seeking behaviours.

Models were fit using the R package ‘brms’ which uses Hamiltonian Markov Chain Monte Carlo to fit hierarchical Bayesian models [19, 20]. We fitted separate models for the B men, K men, B women and K women. The models for women included an additional binary indicator if pregnancy or birth-related codes were observed as described above. As parents were recruited from households, parents are clustered within households. However, as we fitted separate models for men and women, only one parent per household contributes to a single model (the exception being those in same-gender partnerships: less than ten such partnerships were included in the analytical sample, which is less than 0.2% of the households). Only one person per cluster contributes to one analysis (the exceptions were assigned a single $u_i$). Furthermore, it is not assumed that the pattern for men and women is similar, and fitting separate models is easier than fitting a joint model for men and women, including all required interaction terms. It is possible that this approach has reduced efficiency when making comparisons between genders.

Because we used a hierarchical Poisson model with a random intercept for each person, the average count for any age-period-cohort domain is not identical to the median of the counts. We have focussed primarily on the average counts but we note that the ranking of means across domains is similar or identical to the ranking of medians. We illustrate in the Supplementary material that patterns for means and medians provide similar information regarding trends.

### Sensitivity analyses

In a sensitivity analysis, we used 5 fold cross-validation to compare the models with similar ones wherein the smooth term *s* was replaced by (partial pooling) a random term $v_{{C_i},t} \sim N(0,\sigma _v)$ and by (complete pooling) zero. The partial pooling approach assumes that all domain-specific rates vary independently around a global mean, ie it does allow for domain-specific rates but does not encode the knowledge that adjacent cells are more similar to one another. Therefore, it will pull all domain-specific estimates towards the global average, instead of pulling them towards a local average among adjacent cells. Complete pooling can thus be seen as an extreme form of partial pooling, resulting in a flat surface.

Partial pooling and smoothing both result in estimates with lower variability compared to estimating rates in each domain independently (direct estimation), but the lowest variability is achieved by complete pooling: assuming that there is no variation across age, period or cohort. Unfortunately, complete pooling is biased as an estimator for domain-specific rates, unless there is no variability across domains. It can serve as a reference model for both partial pooling and smoothing, as well as for a model that combines both partial pooling and smoothing, ie a model with both terms $s(C_i,t)$ and $v_{C_i,t}$.

To compare models, we use 5-fold cross-validation to calculate the expected log pointwise predictive density (elppd) for each model: we split the data into 5 subsets and set one aside as test data. We refitted the model using 80% of the data as training data and calculate the log of the posterior predictive density for each of the observations in the test data. This procedure was repeated with each of the five subsets as test data, providing a pointwise predictive density for each of the observations in the original data, with elppd defined as the sum of the log pointwise predictive densities. The elppd is a measure of out-of-sample predictive accuracy. The same splits of data into training and tests are used for all models, to ensure fair comparison.

As a second and final sensitivity analysis, we generated an alternative measure for number of episodes of care. Instead of treating health service utilisation sequences on consecutive days as one episode, we extended this to consecutive 30 day periods. We repeated all analyses with this alternative measure as the outcome variable.

### Ethics

At the time of fieldwork, LSAC was conducted in a partnership between the Department of Social Services, the Australian Institute of Family Studies (AIFS), and the Australian Bureau of Statistics. The LSAC study has ethics approval from the AIFS Ethics Committee and written informed consent was obtained from all adults participating in the study. Our study uses data collected for LSAC and has ethics approval from the University of Melbourne Human Research Ethics Committee. Permission to use the data for this study was granted by the data custodians, the National Centre for Longitudinal Data and the Australian Data Archive. The study was carried out in accordance with these ethical guidelines, and the Privacy Act 1988.

## Results

Data linkage provided records for 3006 men and 5251 women. Descriptive statistics for male parents are shown in Table 1. Male parents in the K group are 3 to 4 years older, on average, than male parents in group B. Men’s visit numbers at baseline were 15% higher in the K group than the B group and men had had 35% more visits in wave 7 compared to baseline in the B group and 25% more visits in the K group. In essence, visit numbers increased over time (or age) for men, but with heterogeneous rate of increase. In addition to age, period and cohort effects that could explain this, we observed changes in socio-economic position: educational status increased between baseline and wave 7, while full time employment reduced by only 2 to 3 percentage points. The average equivalised income increased faster than inflation, and is compatible with increased experience.

Descriptive statistics for female parents are shown in Table 2. Female parents in the K group are on average 3.5 years older than female parents in the B group, consistent with the observation for men. The number of GP visits increased with time by 8%, respectively 17%, for female parents in the B group, respectively K group. Female’s visit numbers at wave 7 follow the pattern observed in men, which was consistent with an effect of age: visit numbers were 4% higher in the K group than in the B group. However, at baseline female parents’ visits were 4% lower in the K group than in the B group. As such, visit number increased with time but not necessarily with age for females. The rates of increase appear heterogeneous, and are potentially linked to changes in socio-economic position. These were more pronounced for female parents, and almost certainly this was related to childbirth affecting labour force status. Only 11% of women in group B reported maternity leave at baseline, but another 41% were not in labour force and only 12% reported working full time. At wave 7, 45% worked full time. In group K, full time employment was 21% at baseline and 53% in wave 7. These numbers remain lower than the labour force data for men. The category ‘maternity leave’ does not necessarily capture all births and pregnancies. More information is available from Medicare data: for 71.6% of women in group B, the Medicare data includes at least one birth- or pregnancy-related item in 2003 or 2004, which is much higher than the 14.4% observed for the kinder group, and expected due to the fact that the B group commenced in the first year of the study child’s life. As expected, very few birth- and pregnancy related items were found in the 2016 data.

Comparing descriptive statistics for male and female parents, male parents were on average 2 to 3 years older than female parents. The number of GP visits by men was lower both at baseline and wave 7, for both B and K groups. For both male and female parents, the average number of visits increased with time, and the increase is stronger for men: 35% and 25% increase by wave 7 for groups B and K, while the respective increases in visits for women were 8% and 17%. While the majority of men and women were clustered within households, men were less likely to consent to data linkage. Equivalised household income in consenting male parents was 6% to 11% higher on average than in consenting female parents. In addition, participating men were significantly less likely to be single parents.

**Table 1:** Descriptive statistics for men included in the study. Numerical variables are presented as mean (standard deviation) or median [interquartile range], and categorical variables as count (%)

	b 2004	b 2016	k 2004	k 2016
n	1451	1565	1355	1431
Age	35.0 (5.4)	46.9 (5.6)	38.6 (5.2)	50.5 (5.4)
Cohort	1969	1970	1966	1966
	[1966, 1972]	[1966, 1973]	[1962, 1969]	[1962, 1969]
Australian	1147 (79.0)	1242 (79.4)	1017 (75.1)	1075 (75.1)
Indigenous^a^	11 ( 0.8)	15 ( 1.0)	10 ( 0.7)	12 ( 0.8)
Education				
- 11-	127 ( 8.8)	101 ( 6.5)	154 (11.4)	136 ( 9.5)
- 12	149 (10.3)	109 ( 7.0)	128 ( 9.4)	96 ( 6.7)
- bach+	434 (29.9)	484 (30.9)	401 (29.6)	402 (28.1)
- dipl/cert	736 (50.7)	864 (55.2)	669 (49.4)	785 (54.9)
- NA	5 ( 0.3)	7 ( 0.4)	3 ( 0.2)	12 ( 0.8)
Has Partner	1450 (99.9)	1536 (98.1)	1346 (99.3)	1370 (95.7)
Employment status				
- Full time	1310 (90.3)	1378 (88.1)	1237 (91.3)	1261 (88.1)
- Part time	71 ( 4.9)	90 ( 5.8)	57 ( 4.2)	75 ( 5.2)
- Not in labour force	48 ( 3.3)	62 ( 4.0)	46 ( 3.4)	67 ( 4.7)
- Unemployed	22 ( 1.5)	35 ( 2.2)	15 ( 1.1)	28 ( 2.0)
Income^b^	583	1136	593	1244
	[412, 842]	[784, 1571]	[426, 861]	[873, 1706]
Number of adults	2 [2, 2]	2 [2, 3]	2 [2, 2]	2 [2, 3]
Number of children	2 [1, 2]	2 [1, 3]	2 [2, 3]	2 [1, 2]
GP visits	2 [0, 3]	2 [1, 5]	2 [1, 4]	3 [1, 5]

^a^Indigenous: Aboriginal people, Torres Strait Islander people, or both Aboriginal and Torres Strait Islander people

^b^ Income: total gross household income in Australian dollars, adjusted for the number of people in the household by dividing the annual income by a factor that is equal to one for a single-person household, adding 0.5 for each additional adult and 0.3 for each child (under 15 years)

**Table 2:** Descriptive statistics for women included in the study. Numerical variables are presented as mean (standard deviation) or median [interquartile range], and categorical variables as count (%)

	B 2004	B 2016	K 2004	K 2016
n	2627	2656	2556	2580
Age	32.3 (4.9)	44.3 (5.0)	35.8 (4.8)	47.8 (4.9)
Cohort	1972	1972	1968	1968
	[1968, 1975]	[1968, 1975]	[1965, 1971]	[1965, 1971]
Australian	2104 (80.1)	2128 (80.1)	1982 (77.5)	2002 (77.6)
Indigenous^a^	37 ( 1.4)	36 ( 1.4)	32 ( 1.3)	32 ( 1.2)
Education				
- 11-	287 (10.9)	174 ( 6.6)	417 (16.3)	263 (10.2)
- 12	388 (14.8)	208 ( 7.8)	395 (15.5)	225 ( 8.7)
- bach+	857 (32.6)	835 (31.4)	678 (26.5)	703 (27.2)
- dipl/cert	1094 (41.6)	1436 (54.1)	1066 (41.7)	1384 (53.6)
- NA	1 ( 0.0)	3 ( 0.1)	0 ( 0.0)	5 ( 0.2)
Has Partner	2490 (94.8)	2253 (84.8)	2341 (91.6)	2129 (82.5)
Employment status				
- Full time	307 (11.7)	1195 (45.0)	547 (21.4)	1365 (52.9)
- Part time	858 (32.7)	992 (37.3)	1025 (40.1)	845 (32.8)
- Maternity leave	294 (11.2)	2 ( 0.1)	31 ( 1.2)	0 ( 0.0)
- Not in labour force	1099 (41.8)	395 (14.9)	887 (34.7)	317 (12.3)
- Unemployed	69 ( 2.6)	72 ( 2.7)	66 ( 2.6)	53 ( 2.1)
Income^b^	542	1018	567	1136
	[373, 778]	[667, 1467]	[383, 813]	[754, 1603]
Number of adults	2 [2, 2]	2 [2, 3]	2 [2, 2]	2 [2, 3]
Number of children	2 [1, 2]	2 [1, 3]	2 [2, 3]	2 [1, 2]
GP visits	3 [2, 6]	4 [2, 6]	3 [1, 6]	4 [2, 7]
Any peri- or ante-natal codes	1883 (71.7)	42 ( 1.6)	369 (14.4)	11 ( 0.4)

^a^Indigenous: Aboriginal people, Torres Strait Islander people, or both Aboriginal and Torres Strait Islander people

^b^ Income: total gross household income in Australian dollars, adjusted for the number of people in the household by dividing the annual income by a factor that is equal to one for a single-person household, adding 0.5 for each additional adult and 0.3 for each child (under 15 years)

**Fig. 1:**
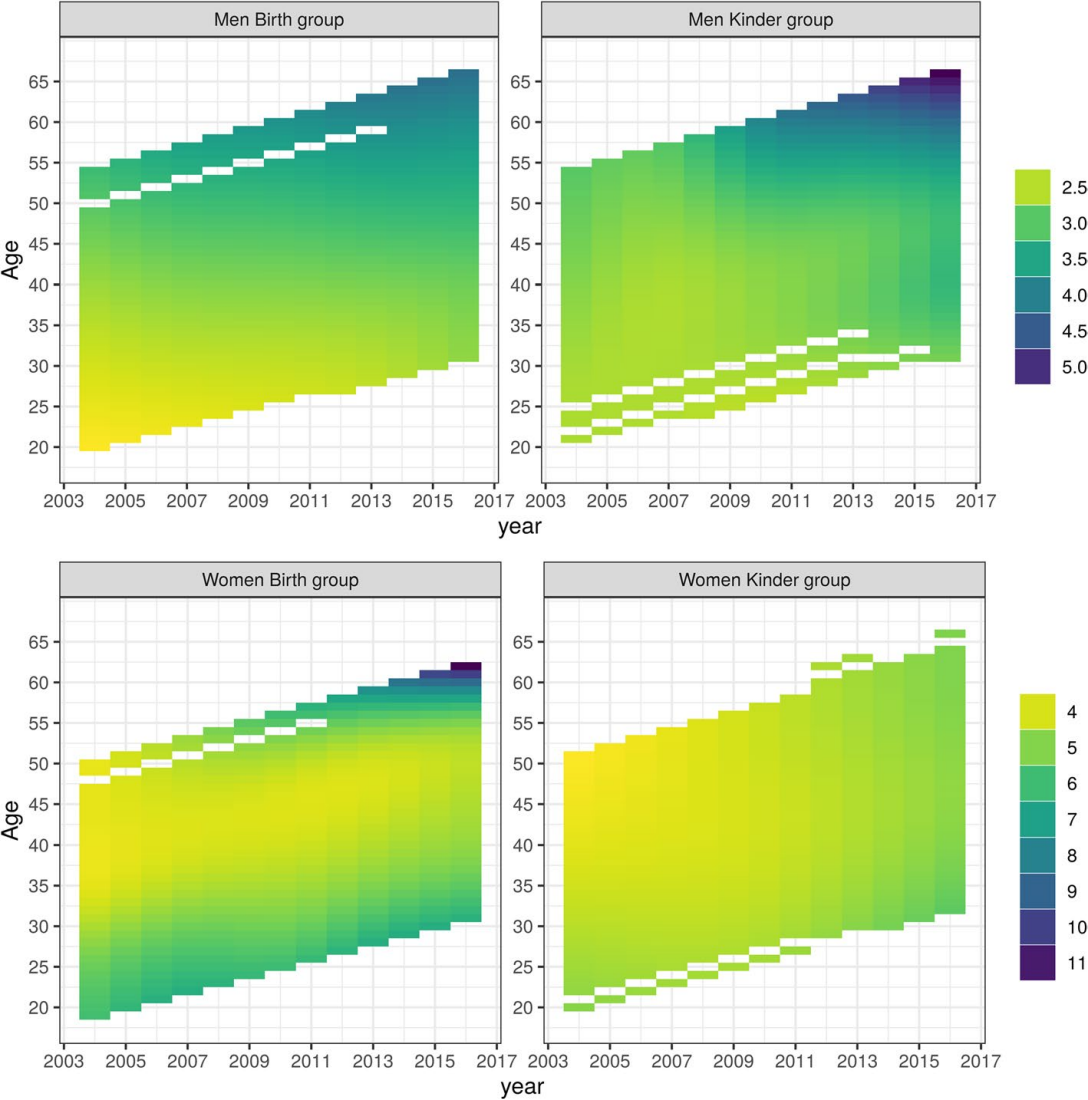
Expected number of annual GP visits of employed parents by age and calendar-year. Top: men. Bottom: women. Left: ‘birth’ group. Right ‘kinder’ group

The top row of Fig. 1 shows the expected number of visits for each age-period-cohort combination for men included in the B and K groups. These were obtained from our models and calculated with the reference group being employed men. The patterns look similar across the two groups, with the expected number of visits ranging from 2.5 to 5 per year. Higher expected counts are observed at later years and higher ages, primarily in men over 50 years old. Weighted by the sample size, these expected counts approximate the observed counts. These expected counts represent the average employed man at specific ages and periods. Even when the median is high, it is possible for a person to have no or few visits. For instance, when the expected number of visits was five for some domains, about one third of men in these domains had no more than two visits. Overall, the patterns across age-period-cohort for expected number of visits were very similar to the expected proportion with 2 or less visits - see Supplementary Material Figs. 3 and 4.

Results for women are displayed in the bottom row of Fig. 1. Here, it was primarily younger women (those who gave birth at 20 to 25 years of age) who visited GPs with higher frequency. For the B group, this occurred throughout the entire period, however we also observed an increase in GP visits with ages over 55 years. This pattern is not perfectly reproduced in the K group, in part this is due to the fact that there were fewer women aged 25 years or less at baseline (2004) and none that were 21 years in 2004, as shown in Supplementary Material Fig. 2. The sample size also changed over time with some participants joining the study later - e.g. through a new marriage - or skipping a wave and thereby providing no information on employment for the period. Because consent to use parents’ linked data was sought in wave seven, analysis was limited to these parents and resembles a complete-case analysis.

**Fig. 2:**
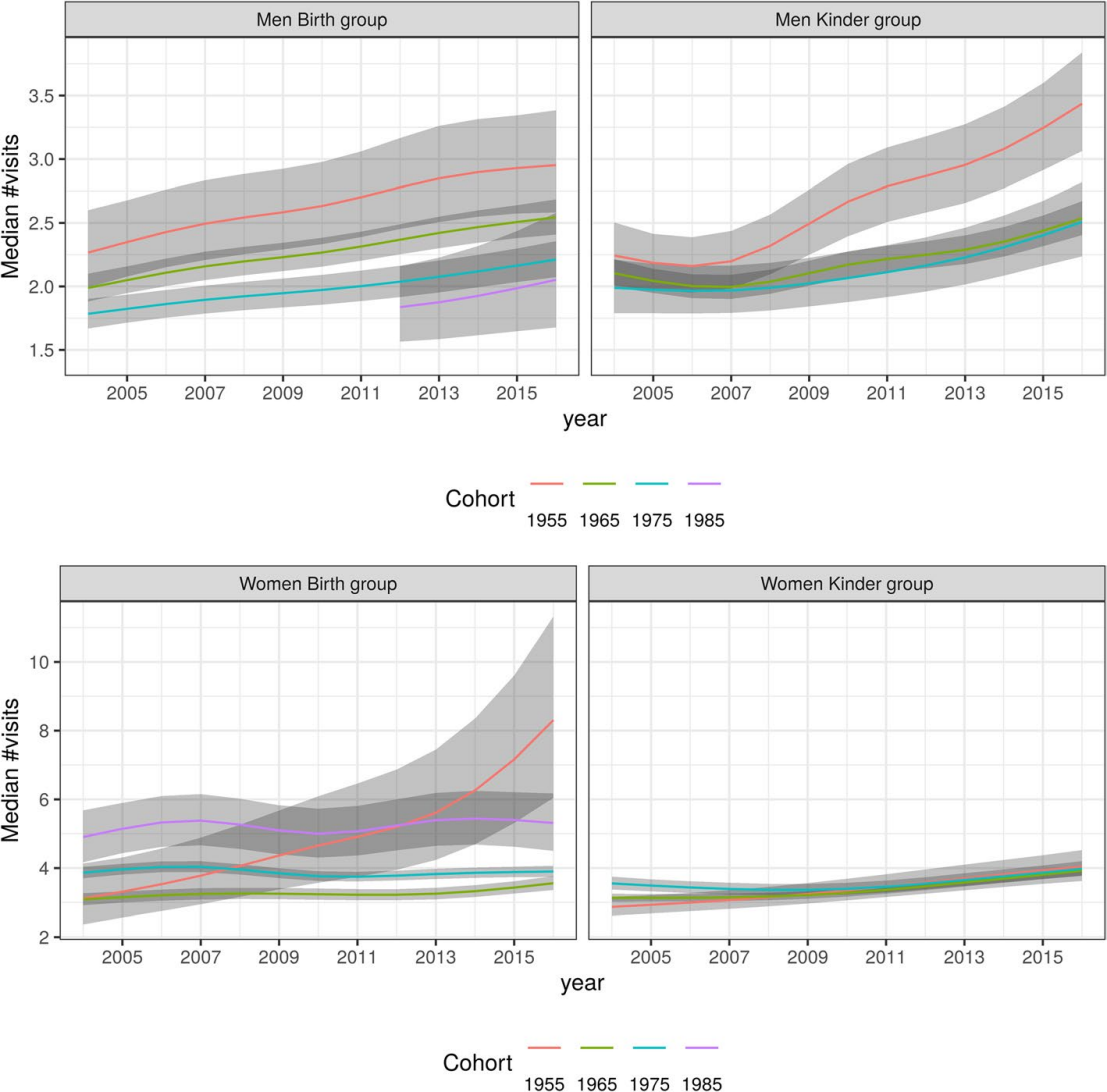
Expected number of annual GP visits for the median employed parent by calendar-year for select birth-year groups. Shaded regions represent 95% pointwise credible intervals. Top: men. Bottom: women. Left: ‘birth’ group. Right ‘kinder’ group

The heatmaps in Fig. 1 do not give an indication of the precision of the estimates. This is better illustrated in Fig. 2 that show the expected number of visits for the median person. For illustrative purposes, we present four birth-years; 1955, 1965, 1975, 1985. Again this graph show that GP visit frequency increased with time or age for men, both in the B and the K group, while the patterns for women differ between the two groups. In the B group women who were born in 1985 had more GP visits, women who were comparatively older at baseline had low visit frequencies at baseline but substantially increased frequencies over time/age. Although the change over time for women in the K group is relatively small, it spans a range larger than that observed for both groups of men. Again, women born in 1955 and 1965 had lower visit frequencies at baseline and these frequencies increased over time. Women from 1975 in the K group had a U-shaped curve, with visit numbers declining across the study before steadily increasing again.

**Fig. 3:**
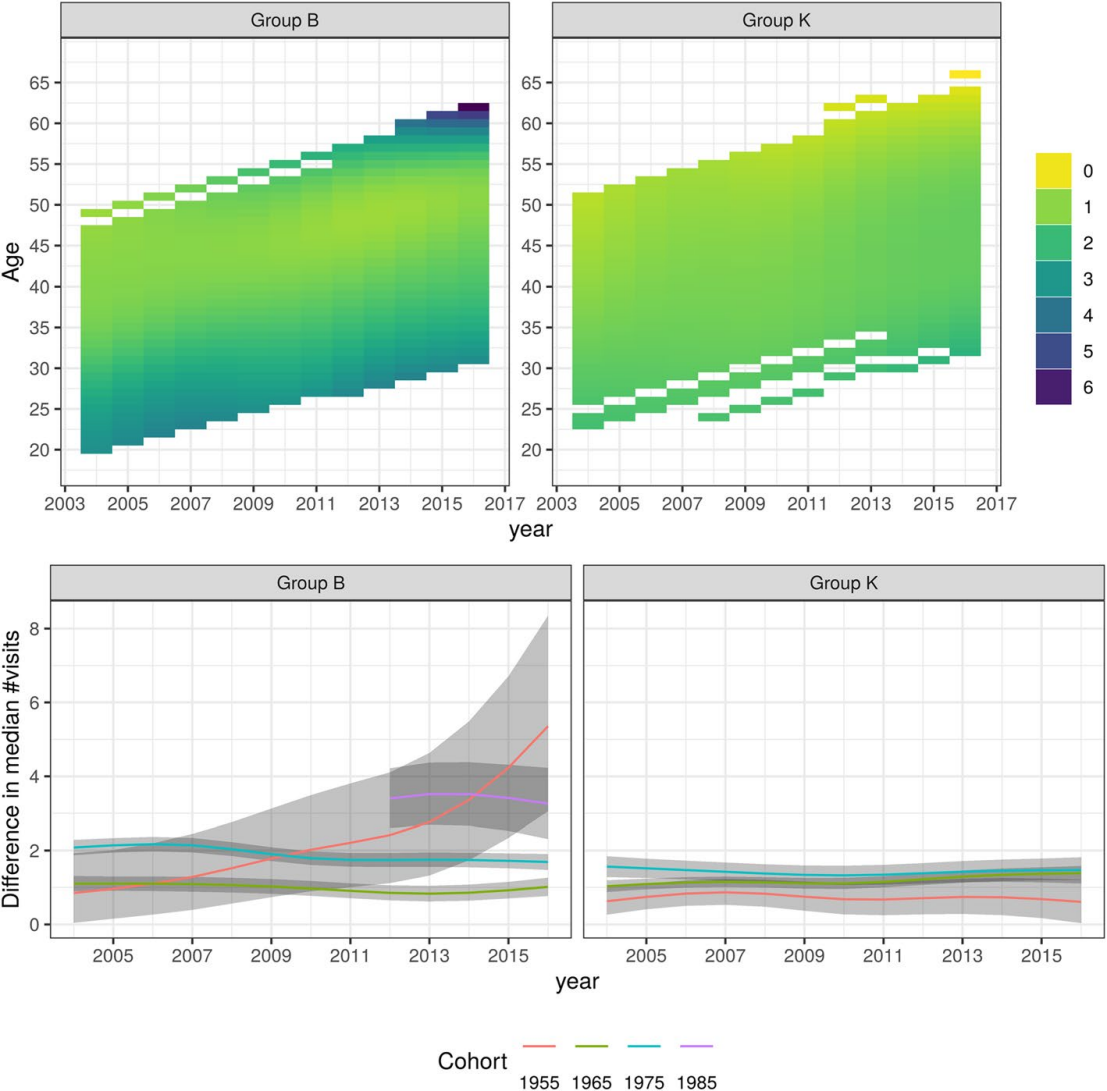
Difference in median number of annual GP visits between female and male parents. Top: heatmap of difference in median by age and calendar-year. Bottom: difference in median by calendar-year, for selected birth-year groups. Shaded regions represent 95% pointwise credible intervals. Left: ‘birth’ group. Right ‘kinder’ group

The gender-specific models show gendered patterns in health service utilisation by age, period and cohort. Figures 1 and 2 show these patterns for the reference person, which we define as a person working full-time, without peri- or antenatal visits, and whose latent $u_i$ is set to zero. Figure 3 displays the contrasts between these reference women and reference men for all age-period-cohort combinations as a heatmap, and for four chosen birth-cohorts. After adjusting for employment and ‘peri-natal’ status, women have more frequent interactions with GPs for almost all age-period-cohort combinations. Less than 3% of the credible intervals include zero, noting that these intervals are neither fully independent nor assumed to represent the same estimand, that only two covariates were adjusted for, and that some of these comparisons are based on small sample sizes.

In our models we adjusted for employment status. For women, we also adjusted for ‘peri-natal’ status. The effect size estimates for these crude additive adjustments are shown in Table 3. There is a small increase (3% to 9%) in GP visits by women in the years surrounding births. Effect size estimates for labour force status are smaller still and the 95% credible intervals include no effect for the contrast not in labour force versus employed for the B group, and for the contrast unemployed versus employed in the K group. Conversely, effect size estimates for men not in labour force compared to employed men are consistent between the two groups at 11% or 12% more visits per year, and consistently include no effect for the contrast of unemployed men versus employed men.

**Table 3:** Effect size estimates and 95% credible intervals for labour force status and ‘peri-natal’ status on the number of GP visits per year, adjusted for age-period-cohort effects and within-person effects. Four models were fitted independently to men and women in the ‘birth’ and ‘kinder’ groups

	Birth group			Kinder group		
	Relative Rate	95% CI		Relative Rate	95% CI	
**Women**	(33652 person-years / 2661 parents)			(32646 person-years / 2590 parents)		
Peri-natal	1.03	1.01	1.05	1.09	1.06	1.12
Not in labour force	1.01	1.00	1.03	1.04	1.02	1.06
Unemployed	1.05	1.01	1.09	1.03	0.99	1.07
**Men**	(19107 person-years / 1570 parents)			(17677 person-years / 1436 parents)		
Not in labour force	1.12	1.06	1.18	1.11	1.05	1.18
Unemployed	1.02	0.95	1.11	0.95	0.88	1.03

### Sensitivity analyses

The patterns observed in these figures are smooth as this is a requirement imposed by the model specified in Eq. 1. One alternative option is ‘partial pooling’ wherein it is assumed that all domain-specific rates vary independently around a global mean. The model with tensor smoothing splines performs better for all groups and there is strong evidence for it performing better in three out of four settings. The difference (standard error) in elppd is 95.2 (33.8), respectively 57.4 (32.0) for women in the B and K groups, and 35.9 (29.8), respectively 85.6 (28.2) for men.

A more complex model is to allow both smooth terms and a random intercept per domain and let the model fit decide how much variation between domains is attributable to an underlying smooth pattern and how much is due to an independently distributed random process. The difference in elppd is small compared to the uncertainty on this metric, indicating both models have similar fit. As shown in Supplementary Material Figs. 5 and 6, patterns obtained from these models are very similar to the patterns obtained from assuming a pure smooth pattern.

Finally, refitting models with the modified measure of GP service use such that 30 day consecutive periods are treated as one episode. This leads to lower average numbers of episodes but the patterns in service use remain similar as shown in Supplementary Material Figs. 7 and 8.

## Discussion

This analysis highlights that the combined effects of age, period and cohort on visits to GPs are different for men and women. The frequency of men’s GP visits increased with age. This pattern is strongly suggestive for the B group and a compatible pattern was observed for men in the K group. In contrast, for women we observed lower frequencies of visits at baseline for those who were comparatively older. In the B group of women who were 25 years or younger at baseline, we observed increased GP visits throughout the study, whereas women aged 45 to 50 years at baseline had initially the lowest and, ultimately, the highest number of visits. For women in the K group there is also a higher visit rate for comparatively younger women at baseline. However, the temporal change in visit rate for these younger women had a u-shaped curve: decreasing in the first four years and then increasing again. Averaged across birth-years, visit numbers for women in the K group increased with time.

Both overall and within each age-period-cohort combination, we observed lower engagement with health services for men relative to women. This is consistent with other research that has shown patterns of lower health service use for men [7, 8]. The pattern for men is comparable between the two groups and likely reflects age effects, with an increase in GP visits with age. Although the descriptive approach does not provide attribution of effects to age, period and cohort effects, the pattern fitted for men in the B group appears to be an effect of ageing; such an explanation is plausible and parsimonious. The fitted pattern for men in the K group shows a stronger contrast for higher ages that is apparent towards the end of the study period, but does not show a pronounced contrast at baseline or younger ages. Noting that men in the K group were four years older on average, there were fewer men in early twenties at baseline included in this group. Conversely, in the K group more men contributed directly to estimates of GP visit numbers for men born in 1950-1960 and the estimates for these birth-years are more precise. This could have driven the different locus of contrast on the graphs for group B and for group K.

The health service patterning is less straightforward for women. In both groups of women there are reversals in the patterning, and in the B group there is a relatively large increase in GP visits by women born about 1955. We note, however, that this pattern is based on relatively few observations and it is not reproduced in the K group. These findings are likely driven by selection into the LSAC: the database intends to be a representative sample of children growing up in Australia. While, by extension, this will approximate representation of parents of these children, that remains substantially different from a representative sample of Australian adults. This deviation from the broader Australian population is apparent in the age structure of parents in 2004. Although LSAC is not restricted to new parents, biological mothers in the B and K groups were healthy enough to give birth at baseline (2004), and 4 years prior to baseline respectively. This selection effect is partially diluted by allowing for new relationships - consent for data linkage was asked in 2016 or 2017 only - but the ‘primary parent’ was usually the mother and women were more likely to respond to the survey than men. The health requirements for giving birth at baseline or 4 years prior, could explain the observed cohort effects among these women; specifically the fact that visits were comparatively high among younger women. We also speculate that socio-economic position (SEP) may exert an influence here. SEP is a key determinant of health and well-being [21] (and health-seeking behaviour), and in many Western democracies (including Australia), SEP is also associated with the age at which women give birth [22]; higher socio-economic position is associated with longer participation in education and vice versa; that is, women who study longer tend to delay marriage and parenthood.

Selection effects of socio-economic status are also apparent when comparing the distribution of reported income by male and female participating parents (noting that fewer men participated than women), with the average equivalised family income for participating men being 6% to 11% higher than the average among participating women. We also observe that about 90% of participating men reported working full time, whereas for women this varied between 11.8% and 52.9% and was lower at baseline than at the end of follow-up. Women seeking and securing employment likely drives the change in income over time. Furthermore, it implies changes in life-style and division of carer responsibilities. We have attempted to capture this by including employment status as an independent variable in our models, however, we did not include employment status of the partner.

In terms of the implications of this analysis, the pattern observed here can parsimoniously be explained purely as an effect of ageing with negligible or non-existent changes in service engagement. Conversely, cohort and period effects - and possibly interactions between those - would be indicative of normative and behavioural changes in men’s health service engagement. The fact that there is little evidence of any changes unrelated to ageing suggests no such changes, albeit the study period is short compared to the range of ages and birth-years and power to detect period effects is therefore lower. If men’s health service engagement is currently insufficient for their current health needs, then there is no evidence it will improve - or worsen - in the near future. When considering the implications of this study, it is important to acknowledge that the GP visits included in this analysis only reflect health service use, and not diagnosis, health condition or health service need. It is therefore impossible to determine whether the statistics reflect an engagement with health services that is adequate and sufficient for the health needs of the population, or whether it reflects reluctant or late engagement. Nonetheless the overall pattern of lower engagement for men needs further investigation to assess the extent to which it corresponds with rates of hospitalisation and mortality - as noted, research elsewhere has shown a pattern of lower engagement with health services and correspondingly higher rates of hospitalisation and mortality for men [9].

There are some important strengths and limitations of this analysis. Selection and attrition bias are important limitations. The design of LSAC is less conventional than other linkage studies, with request for data linkage being sought at wave seven. Although LSAC is intended to be a representative sample of children in Australia, it does not automatically provide a representative sample of adults (because parents with dependent children tend to comprise a narrower age range than the population as a whole). Indeed, a large fraction of participating adults were aged between 30 and 39 years of age at study commencement. As there were few participants below 20 years or over 55 years at baseline, they were excluded from this study. In addition to affecting sample size, this limits generalisability to the general adult population. As mentioned before, some of the observed patterns in women are potentially due to selection on health and socio-economic position.

A further limitation is that our approach assumed an additive effect (on the log-scale) of time-invariant unobserved within-person variables such as genotype or health-seeking behaviour. This does not address the fact that behaviour could be modifiable over time, and overall health can change particularly with onset of chronic conditions but also via lifestyle changes. It is also important to note that the observed patterns must be interpreted as hypothesis-generating; no causal assumptions are made nor can they be derived from this approach. Although we did calculate effect size estimates for labour force status and ‘peri-natal’ status, it is not clear whether these associations are causal, reverse causes (i.e. job loss due to poor health) or have a common cause. Furthermore, labour force and ‘peri-natal’ status were also modelled as additive effects. This allowed a larger sample size than restricting to parents who were continuously working full-time. Should true APC patterns differ among employment strata, the estimated patterns would be equivalent to a weighted average of these patterns. Especially for men, the dominant category in our sample is full-time employment.

Finally, we acknowledge that there may have been some misclassification of gender in this analysis. Gender is a complex social construct, and a person’s gender identify does not necessarily align with sex assigned at birth [23].

The main strengths of our approach are the use of longitudinal data and the use of objective linked measures of health service usage. Our analytical approach enabled us to observe significant variation in the underlying rates of service usage between participants after adjusting for differences across age, cohort, period and employment status, and taking into account the pure noise of a count process. This represents a significant advancement on cross-sectional approaches and repeated cross-sectional designs, which cannot achieve this regardless of sample size. A further strength is that the smoothing technique we employed accommodated small sample sizes within each age-period combination of one life-year by one calendar-year. This was illustrated by using cross-validation as a tool to compare model-fit, noting that 5-fold cross-validation effectively reduces the sample size of the training set by 20%.

## Conclusions

This examination of health service engagement, assessed in terms of GP visits, demonstrates differences in health service utilisation between male and female parents at all age-period-cohort combinations, taking into account employment status. The results align with other research demonstrating lower health service use for men. The observed patterns for men suggest age-effects - that is effects of ageing. Negligible change in health service utilisation over the study period indicates an absence of period or cohort effects. This highlights a need for more research to examine the extent to which this level of health service use among Australian men meets men’s health needs, as well as barriers and enablers of health service engagement for men.

## Supplementary Information

**Additional file 1.**

## Data Availability

The data that support the findings of this study are available from the Australian Data Archive but restrictions apply to the availability of these data, which were used under license for the current study. Data access can be requested here: https://dataverse.ada.edu.au/dataset.xhtml?persistentId=doi:10.26193/F2YRL5. The source code to generate tables and figures is available on https://bitbucket.org/Koen_Simons/lsac_apc_serviceuse/src/master/.

## Abbreviations

APC: Age-Period-Cohort
elppd: expected log pointwise predictive density
GP: General Practitioner
LSAC: ‘Growing up in Australia: The Longitudinal Study of Australian Children’
MBS: Medicare Benefit Schedule
SEP: socio-economic position
