# Supplementary material for: Age and gender patterns in health service utilisation: Age-Period-Cohort modelling of linked health service usage records
Source: BMC Health Serv Res. 2023 May 12;23:480. doi: 10.1186/s12913-023-09456-x (PMC10176675; doi:10.1186/s12913-023-09456-x)

AGE AND GENDER PATTERNS IN HEALTH SERVICE UTILISATION: APC MODELLING OF LINKED HEALTH SERVICE USAGE RECORDS

ADDITIONAL FILES

Koen Simons
Centre for Epidemiology and Biostatistics
Melbourne School of Population and Global Health
University of Melbourne, AU
koen.simons@unimelb.edu.au

Owen Bradfield
Centre for Health Policy
Melbourne School of Population and Global Health
University of Melbourne, AU
owenmb@student.unimelb.edu.au

Matthew J Spittal
Centre for Mental Health
Melbourne School of Population and Global Health
University of Melbourne, AU
m.spittal@unimelb.edu.au

Tania King
Centre for Health Equity
Melbourne School of Population and Global Health
University of Melbourne, AU
tking@unimelb.edu.au

May 9, 2023

1 MBS Codes

Table S1: Selected MBS item numbers and total occurrence for (left) GP visits and (right) ante and perinatal care.

MBS item number	n
3	9639
23	334056
36	51557
44	5578
597	1120
599	190
5000	143
5020	18551
5023	354
5028	2
5040	1786
5043	36
5060	157

MBS item number	n	MBS item number	n
16400	200	16512	3
16401	133	16514	678
16404	144	16515	3
16406	2	16518	8
16500	29603	16519	1708
16501	6	16520	10
16502	175	16522	644
16504	4	16525	4
16505	32	16564	28
16508	103	16567	7
16509	108	16570	1
16511	8	16573	3

2 Participant flow chart

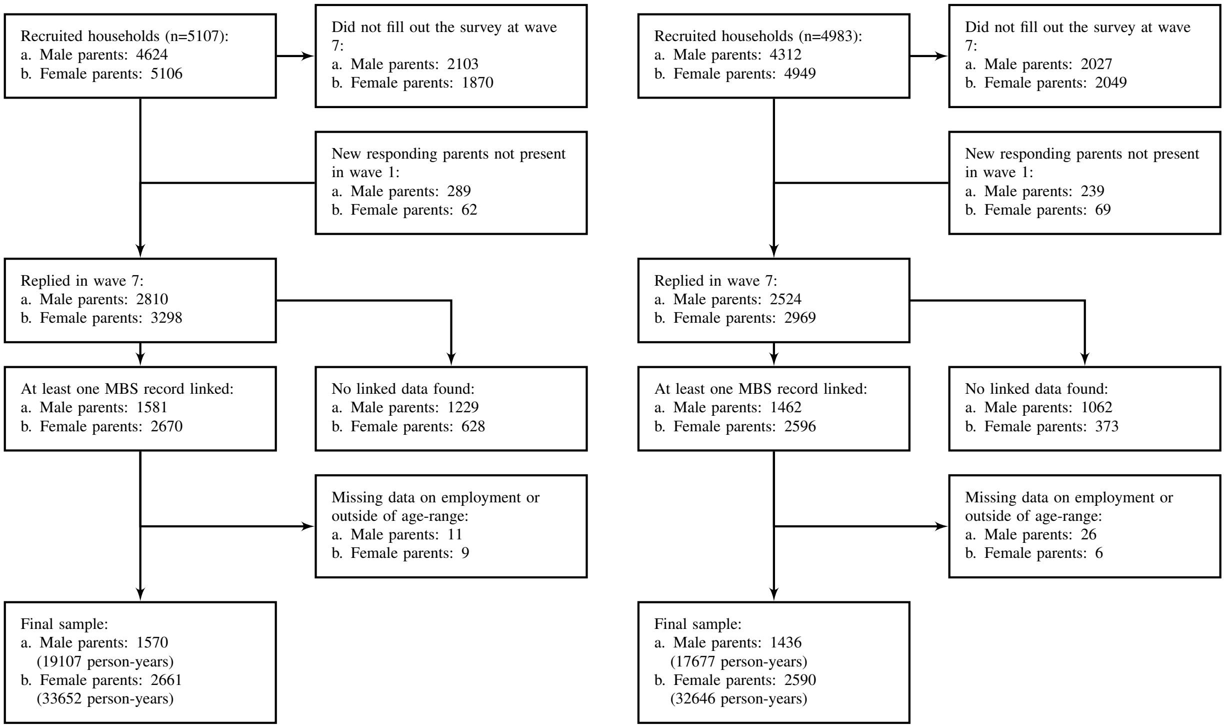

Figure S1: Participant flow chart. Left: ‘birth’ group. Right: ‘kinder’ group.

3 Sample sizes

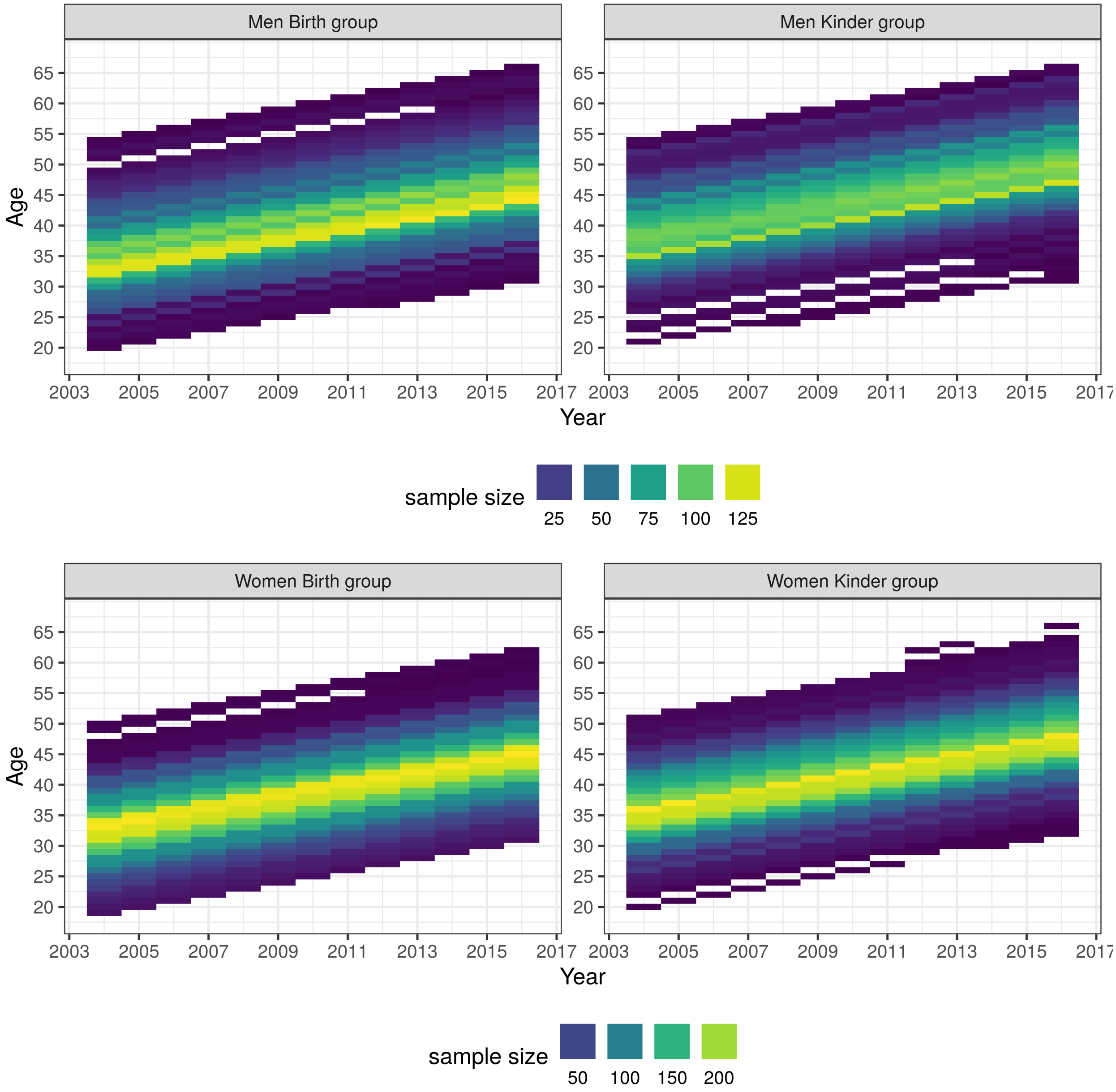

Figure S2: **Sample size of parents by age and calendar-year. Top: men. Bottom: women. Left: 'birth' group. Right 'kinder' group.**

4 Expected counts versus proportions with at least two visits

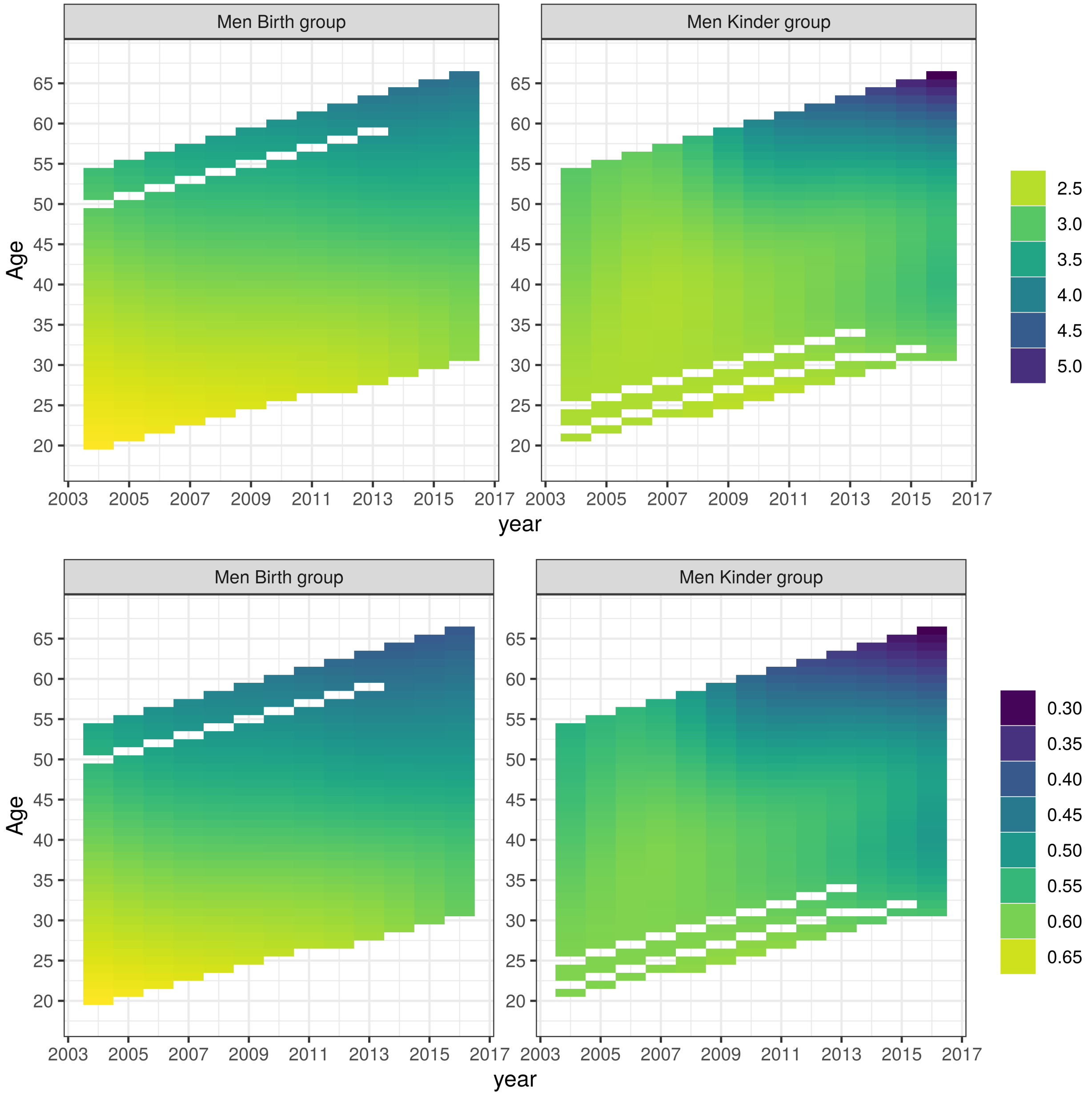

Figure S3: **Comparison of patterns: expected count of visits versus proportion with at most two visits. Top: expected number of annual GP visits of employed men by age and calendar-year. Bottom: expected proportion of employed men with 2 or less GP visits in a given year. Left: 'birth' group. Right 'kinder' group.**

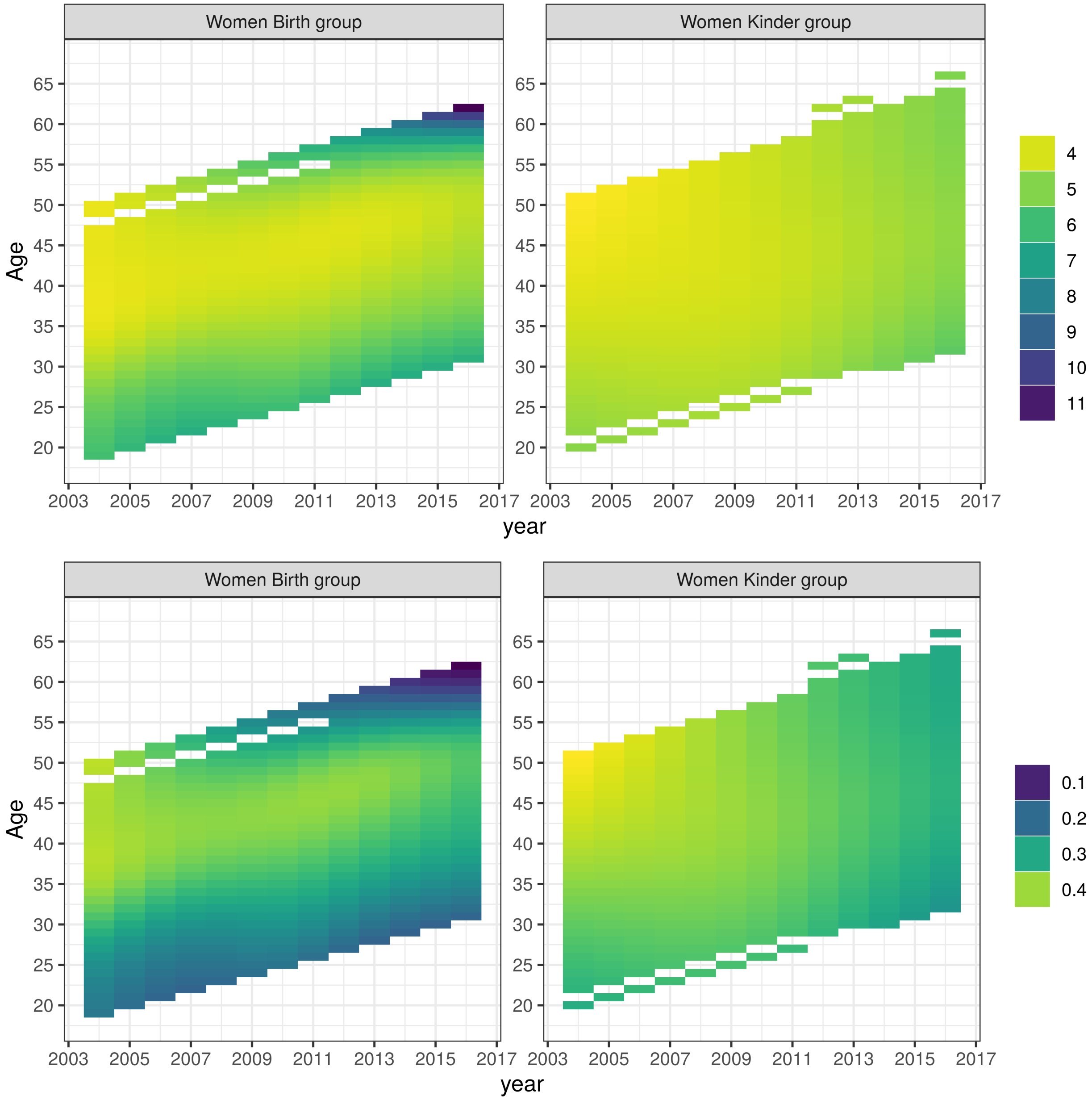

Figure S4: **Comparison of patterns: expected count of visits versus proportion with at most two visits. Top: expected number of annual GP visits of employed women by age and calendar-year. Bottom: expected proportion of employed women with 2 or less GP visits in a given year. Left: 'birth' group. Right 'kinder' group.**

5 Sensitivity analysis: model

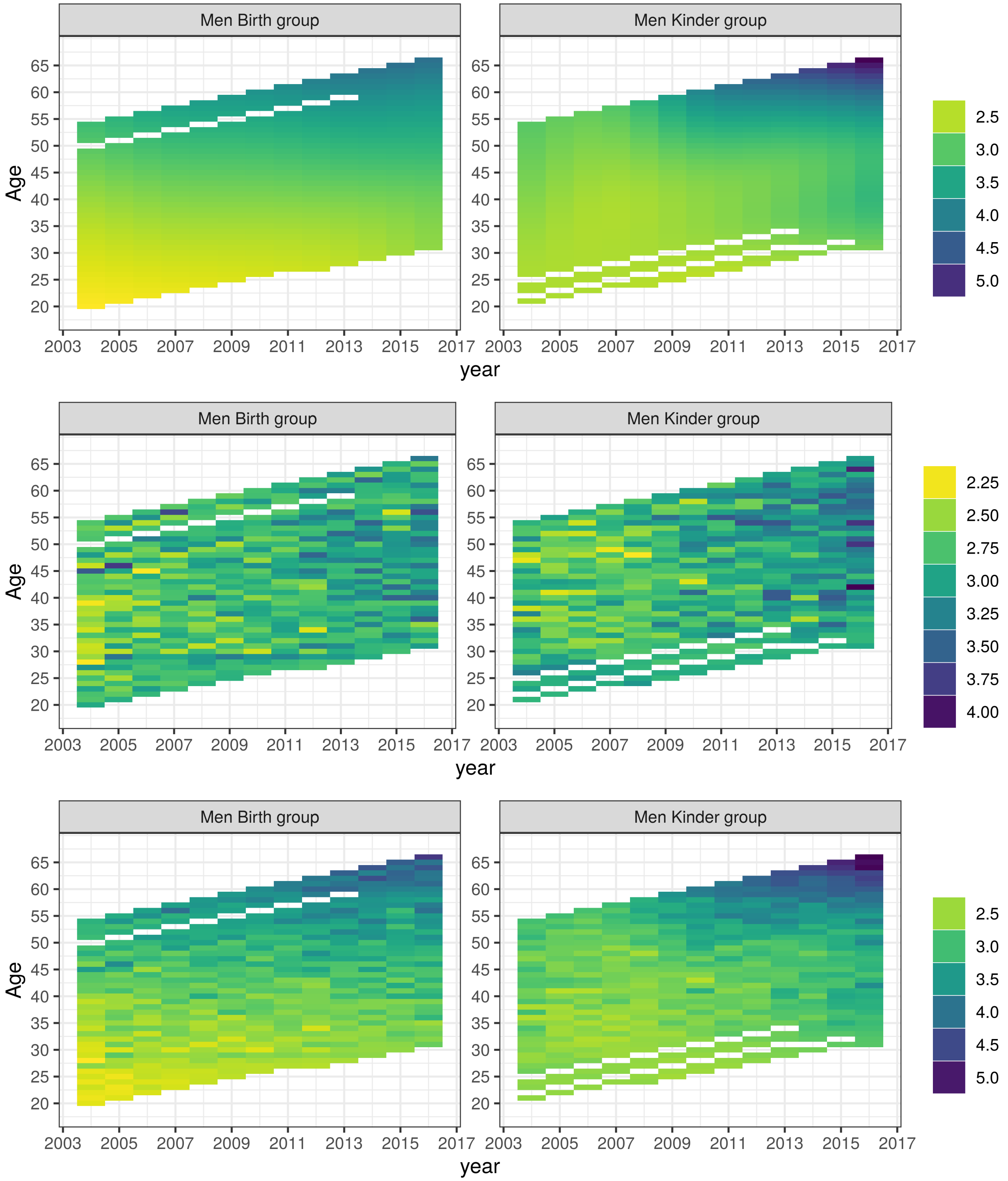

Figure S5: **Sensitivity Analysis: comparison with partial pooling. Expected number of annual GP visits of employed men by age and calendar-year. Left: 'birth' group. Right 'kinder' group. Models used to derive patterns from top to bottom: smooth(APC), partial pooling (random intercept for APC), smooth(APC) + random(APC)**

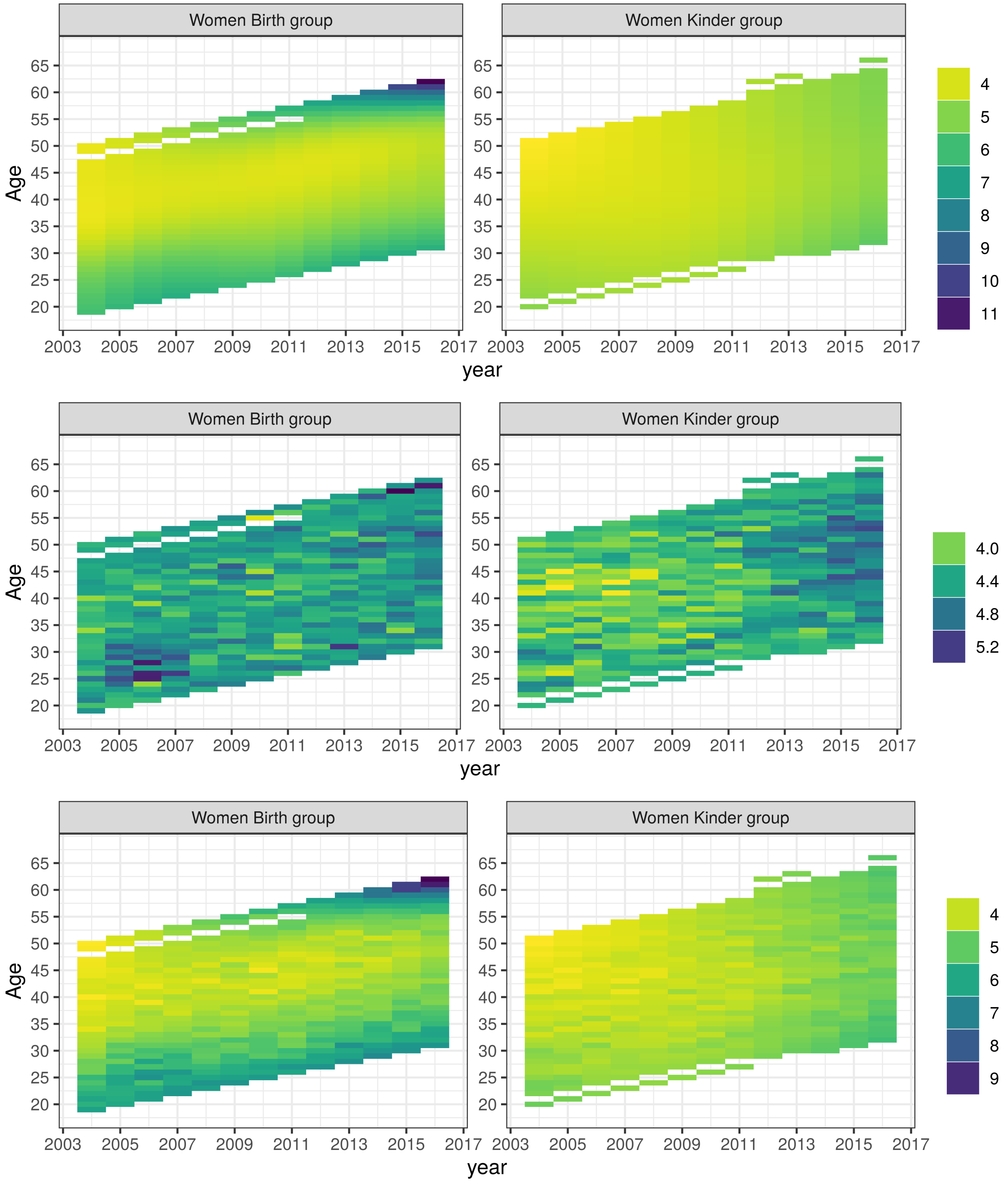

Figure S6: **Sensitivity Analysis: comparison with partial pooling. Expected number of annual GP visits of employed women by age and calendar-year. Left: 'birth' group. Right 'kinder' group. Models used to derive patterns from top to bottom: smooth(APC), partial pooling (random intercept for APC), smooth(APC) + random(APC)**

6 Sensitivity analysis: outcome definition

Table S2: Effect size estimates and 95% credible intervals for labour force status and 'peri-natal' status on the number of GP visits per year, *with at least 30 days between visits*, adjusted for age-period-cohort effects and within-person effects. Four models were fitted independently to men and women in the 'birth' and 'kinder' groups.

women	birth group			kinder group		
	Relative Rate	95% CI		Relative Rate	95% CI	
	(33652 person-years / 2661 parents)			(32646 person-years / 2590 parents)		
peri-natal	1.04	1.01	1.06	1.07	1.03	1.11
not in labour force	1.01	0.99	1.03	0.99	0.97	1.01
unemployed	1.00	0.95	1.05	1.02	0.97	1.07

men	birth group			kinder group		
	Relative Rate	95% CI		Relative Rate	95% CI	
	(19107 person-years / 1570 parents)			(17677 person-years / 1436 parents)		
not in labour force	1.08	1.00	1.17	1.09	1.01	1.18
unemployed	1.01	0.90	1.12	1.03	0.92	1.14

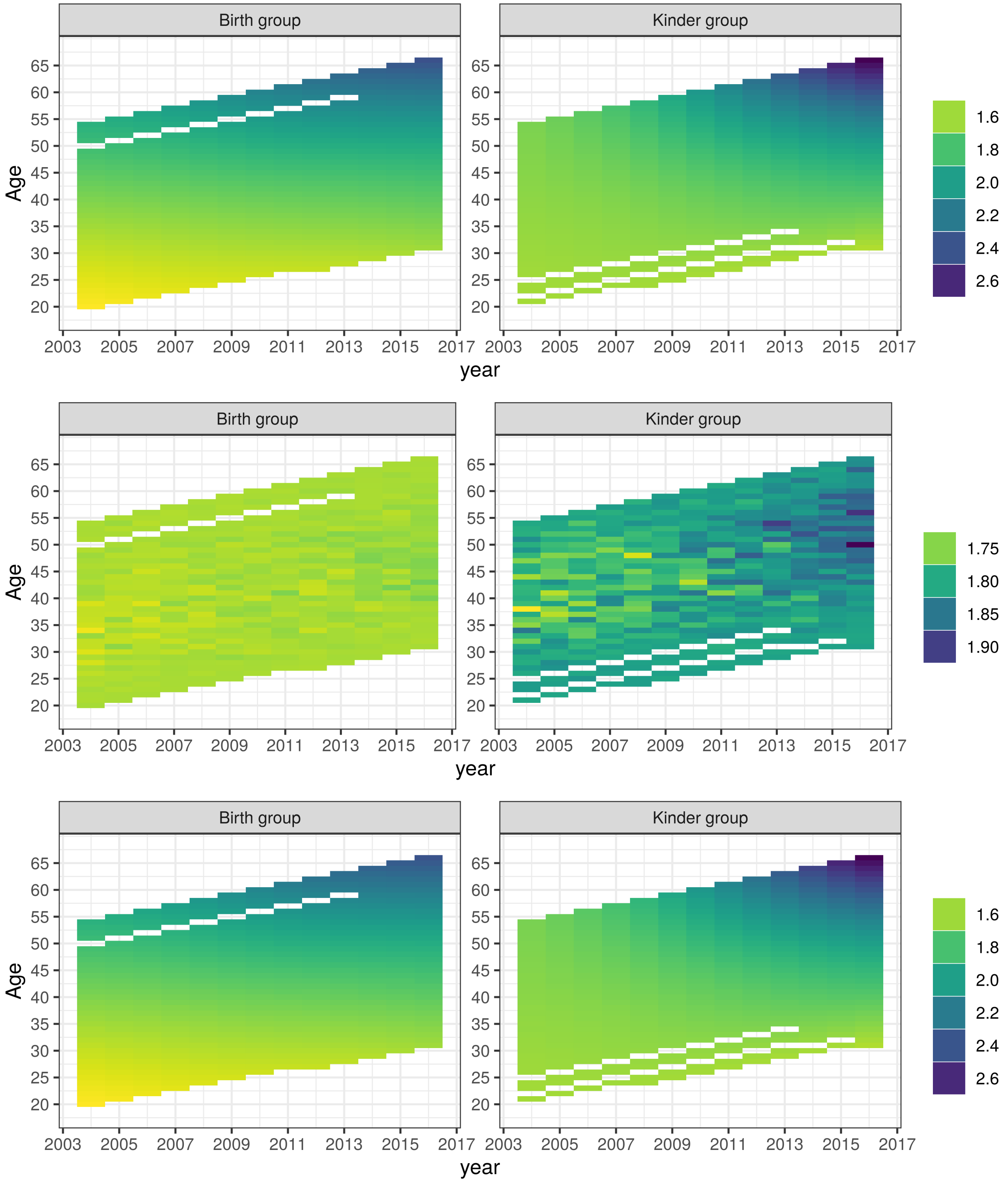

Figure S7: **Sensitivity Analysis: alternative measure of health service use. Expected number of annual GP visits *with at least 30 days between visits*, for employed men by age and calendar-year. Left: 'birth' group. Right 'kinder' group. Models used to derive patterns from top to bottom: smooth(APC), partial pooling (random intercept for APC), smooth(APC) + random(APC).**

Figure S8: **Sensitivity Analysis: alternative measure of health service use. Expected number of annual GP visits *with at least 30 days between visits*, for employed women by age and calendar-year. Left: 'birth' group. Right 'kinder' group. Models used to derive patterns from top to bottom: smooth(APC), partial pooling (random intercept for APC), smooth(APC) + random(APC).**

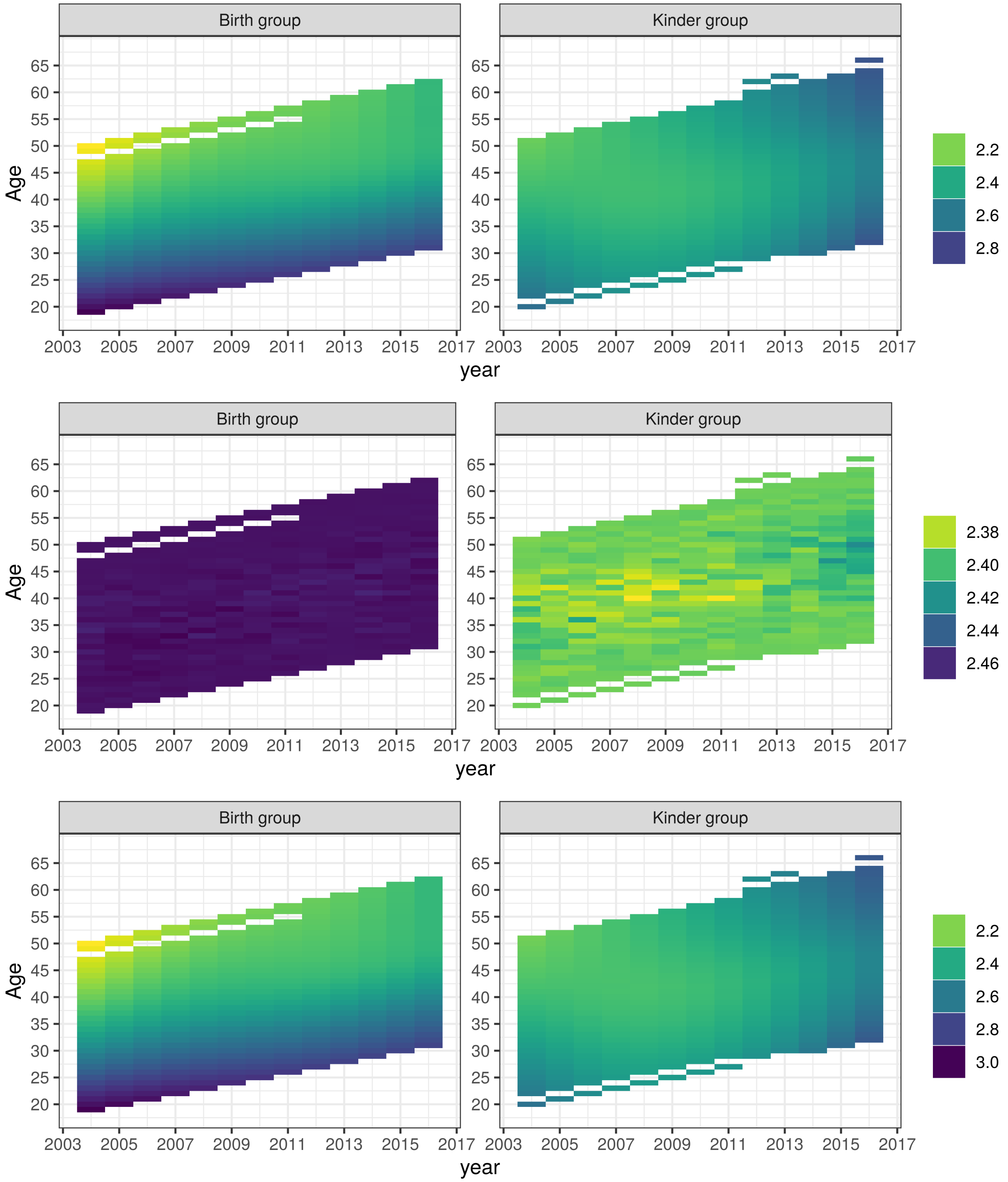

Supplement: Supplementary file 1 — Additional file 1. [file 12913_2023_9456_MOESM1_ESM.pdf]
